# Surgical management of orbital wall fractures in juveniles: a European multicenter study

**DOI:** 10.1007/s00068-026-03344-9

**Published:** 2026-10-14

**Authors:** Paolo Boffano, Anssi Lahtinen, Matteo Brucoli, Muhammad Ruslin, Andrea Melle, Panagiotis Stathopoulos, Dimitra Maniati, Emil Dediol, Boris Kos, Adam Michcik, Adam Polcyn, Thomas Starch-Jensen, Ivana Mijatov, Branislav V. Bajkin, Annabelle Cailleaux, Eugénie Bertin, Christophe Meyer, Aurélien Louvrier, Andrii V. Kopchak, Tetiana A. Pavlychuk, Sergi Herrera I Nogués, Ana Martínez-Carrillo, Juan Carlos de Vicente, Linas Zaleckas, Rūta Rastenienė, Augustinas Stasiūnas, Jan Naberoj, Luka Prodnik, Tadej Dovsak, Johanna Snäll

**Affiliations:** 1 Department of Health Sciences, University of Eastern Piedmont, Novara, Italy; 2 Department of Oral and Maxillofacial Surgery, Faculty of Dentistry, Hasanuddin University, Makassar, Indonesia; 3 Department of Oral and Maxillofacial Diseases, University of Helsinki, Helsinki University Hospital, Helsinki, Finland; 4 First General Hospital of Athens K.A.T, Athens, Greece; 5 Department of Maxillofacial Surgery, University Hospital Dubrava, Zagreb, Croatia; 6 Department of Maxillofacial Surgery, Faculty of Medicine, Medical University of Gdansk, Mariana Smoluchowskiego 17, Gdansk, 80-214 Poland; 7 Department of Oral and Maxillofacial Surgery, Aalborg University Hospital, Aalborg, Denmark; 8 Faculty of Medicine, Clinic for Maxillofacial Surgery, University of Novi Sad, University Clinical Center of Vojvodina, Novi Sad, Serbia; 9 Faculty of Medicine, University of Novi Sad, Dental Clinic of Vojvodina, Novi Sad, Serbia; 10 Université Marie et Louis Pasteur, SINERGIES (UR 4662), Besançon, F-25000 France; 11 Chirurgie maxillo-faciale, stomatologie et odontologie hospitalière, Université de Franche-Comté, CHU Besançon, Besançon, F-25000 France; 12 Department of Maxillo-Facial Surgery, Bogomolets National Medical University, Kyiv, Ukraine; 13 Maxillofacial Department, Hospital Universitario Central de Asturias from Oviedo, Asturias, Spain; 14 Institute of Dentistry, Faculty of Medicine, Vilnius University, Vilnius, Lithuania; 15 University Medical Centre Ljubljana, Ljubljana, Slovenia

**Keywords:** Pediatric, Orbital fracture, Complication, Surgery, Management

## Abstract

**Introduction:**

Isolated orbital floor fractures in juveniles are a challenge, as children and adolescents may experience diplopia and limited ocular motility, even with little to no periorbital edema or visible signs of injury to the eye. The aim of the present study is to assess the demographic and clinical variables, and the outcomes of pediatric patients affected by isolated orbital wall fractures at several European oral and maxillofacial surgery centers.

**Methods:**

Pediatric patients (less than 18 years) with hospitalized and surgically treated for unilateral isolated orbital blow out fractures of the orbital floor and/or the medial wall were included. The following data were recorded for each patient: gender, age, etiology of trauma, site of orbital wall fracture(s), timing of intervention, surgical approach, orbital reconstruction material/implant, post-operative complications

**Results:**

A total of 51 pediatric patients (45 boys, 6 girls) with a mean age of 12.1 years were included. Most patients (43 children, 84.3%) presented with an isolated orbital floor fracture. Clinically, the most frequently observed sign was diplopia / double vision (43 patients, 84.3%). More than two thirds (68.6%) of cases were surgically treated within the first two days from injury. In 6 patients (11.8%) one or more persistent postoperative disturbances were found six months postoperatively, including diplopia in 3 subjects (5.9%), infraorbital nerve paresthesia in 2 patients (3.9%), and enophthalmos in 2 patients (3.9%).

**Conclusions:**

The rarity of pure orbital wall fractures in pediatric patients makes it difficult to establish uniform evidence-based guidelines for their management. These injuries must also be recognized promptly, as surgical intervention may be required at a very early stage. A reasoned and individualized choice of the optimal surgical approach appears to be the most appropriate strategy for this specific type of facial fracture in the pediatric population.

**Clinical trial number:**

Not applicable.

## Introduction

Isolated orbital floor fractures in juveniles are a challenge, as children and adolescents may experience diplopia and limited ocular motility, even with little to no periorbital edema or visible signs of injury to the eye. The term “white-eyed’ blowout fractures have, therefore, often been used to characterizes this fracture [1–14].

Consequently, orbital soft tissues including orbital fat and extraocular muscles may easily be incarcerated causing diplopia, which is often associated with nausea, vomiting, and bradycardia, that are signs of the oculocardiac reflex.

In fact, the growth phase of the pediatric population affects the fracture pattern and associated complications in juveniles, as the immature pediatric facial skeleton provides high resistance to fracture. Despite the low frequency of pure orbital wall fractures within the whole pediatric facial injuries, they may determine severe complications and long-term morbidity [1–8, 10–12].

High rates of entrapment have been reported in the literature following pediatric orbital floor fractures [1–10]. Due to the elastic bone structure, the fracture gap in correspondence of the orbital wall (and in particular in correspondence of the orbital floor) may open during trauma and snap back, thereafter, determining the high risk of entrapment of orbital soft tissue such as orbital fat and/or muscles. Hence the term “orbital trapdoor fracture”.

Thus, incarceration of orbital soft tissue may cause tissue ischemia with a risk of necrosis, scarring, and functional impairments with long-term complications including persistent diplopia and restricted extraocular motility. Therefore, early diagnostics are of paramount importance. Deficiencies have been observed in recognizing orbital injuries in children, as the symptoms can be highly variable.

The timing of surgery for pediatric orbital wall fractures has been highly debated. In fact, some studies have recommended surgery as soon as possible whereas other studies have proposed differentiated protocols with different timing of surgery [1–9]. On the other hand, soft tissue entrapments may vary in nature, and specifically the identification of extraocular muscle incarceration is crucial, as it necessitates prompt surgical intervention by an experienced surgeon to achieve an optimal outcome.

However, studies with large patient samples assessing orbital wall fractures in juveniles are scarce [1–10]. 

The aim of the present study is, therefore, to assess the demographic and clinical variables, the diagnosis, the management, and the outcomes of pediatric patients affected by isolated orbital wall fractures at several European oral and maxillofacial surgery centers, in order to minimize selections biases and provide information about the epidemiology, etiopathogenesis, current trends in the treatment, and the outcomes of isolated orbital wall fractures across Europe.

## Materials and methods

This study utilized a systematic computer-assisted database that allowed recording data on all hospitalized and treated cases of isolated orbital wall fractures at participating surgical units across Europe between January 1st, 2015, and December 31st, 2024.

Helsinki Declaration guidelines were followed, according to local laws, in addition to STROBE criteria for observational studies. Ethical approval was obtained by each center if needed, according to local laws.

Pediatric patients (less than 18 years) with hospitalized and surgically treated for unilateral isolated orbital blow out fractures of the orbital floor and/or the medial wall were included. “Isolated” fractures were defined as fractures not extending to orbital rims and not making part of an homolateral orbito-maxillary-zygomatic fracture (isolated orbital fractures in association with contralateral orbito-maxillary-zygomatic fractures were included).

Exclusion criteria were as follows: (i) fracture extending to orbital rim(s) (ii), retrobulbar hemorrhage/hematoma at diagnosis requiring surgical treatment, (iii) secondary reconstruction cases, (iiii) presence of comorbidities or substance abuse, (iiiii) patients with repeated injuries.

The following data were recorded for each patient: gender, age, etiology of trauma, site of orbital wall fracture(s), concomitant facial fractures, concomitant body injuries, associated clinical signs and symptoms, timing of intervention, surgical approach, orbital reconstruction material/implant, post-operative complications, length of hospital stay, and outcome.

The following categories of the etiology were considered: motor vehicle accident, falls on stairs or from height, ground-level falls, assault, sport injury, work injury, war related injury, bicycle injury, E-scooter injury, and other causes.

The eventually associated facial fractures were determined from computed tomography scans at admission to the hospital and classified as fractures of the mandible, orbital-zygomatic-maxillary complex (MZO), orbit, nose, Le Fort, frontal sinus, and naso-orbital-ethmoid (NOE) fracture.

The eventually associated concomitant body injuries were classified as orthopedic, brain, spine, ocular, thoracic, and abdominal injuries.

The primary outcome variable was the presence or absence of persistent postoperative disturbances / complications after a six months postoperative observation period, including decreased visual acuity, diplopia, infraorbital nerve paresthesia, malposition of the globe (i.e., enophthalmos or hypophthalmos ≥ 2 mm).

Data was recorded in Microsoft Excel (Microsoft Inc., Redmond, WA, USA). Statistical analysis was performed using SPSS software (IBM Corp., Armonk, New York, US). Categorical variables were analyzed using standard statistical methods such as count, frequency tables, and proportion analyses. Statistical significance for categorical variables was determined using the chi-square test or, if the sample was too small, the Fisher exact test. The Mann–Whitney U test was used to compare continuous variables, and *p* < 0.05 was considered statistically significant.

## Results

A total of 51 pediatric patients (45 boys, 6 girls) diagnosed with unilateral isolated orbital blow out fractures of the orbital floor and/or the medial wall fulfilling the inclusion criteria were included. The mean age of the study population was 12.1 years (median 13 years; SD 3.9 years; range 4–17 years).

As for the etiology of trauma, the most frequent cause of injury was sports accidents (17 patients, 33.3%), followed by ground – level falls with 10 patients (19.6%), assaults (8 patients, 15.7%), and bicycle accidents (6 patients, 11.8%) (Table 1).

**Table 1:** Descriptive statistics of 51 minor patients with pure orbital fractures

Age (years)		
mean	12.1 (SD 3.9)	
median	13	
range	4–17	
	**n total**	**% of 51**
**Sex**		
Male	45	88.2%
Female	6	11.8%
**Etiology**		
Sports	17	33.3%
Ground-level falls	10	19.6%
Assaults	8	15.7%
Bicycle	6	11.8%
Falls on stairs or from height	5	9.8%
Motor vehicle accidents	2	3.9%
Works	1	2.0%
E-scooter	1	2.0%
Other	1	2.0%
**Pure orbital fracture type**		
Orbital floor	43	84.3%
Orbital floor + Orbital medial wall	5	9.8%
Orbital medial wall	3	5.9%
**Concomitant facial fractures**		
Yes	5	9.8%
**Concomitant body injuries**		
Yes	6	11.8%

Concomitant body injuries were observed in 6 patients (11.8%). Most frequently observed concomitant injuries were traumatic brain injuries (3 patients), and orthopedic injuries (2 patients).

In 5 patients (9.8%), one or more associated facial fractures were diagnosed together with isolated orbital wall fractures. Nasal fractures were the most frequently observed associated fracture type in three patients.

As for pure orbital fracture types (Table 1), most patients (43 children, 84.3%) presented with a isolated orbital floor fracture, whereas 5 patients (9.8%) presented with a combined orbital floor and orbital medial wall fracture, and 3 subjects (5.9%) had an orbital medial wall fracture.

Clinically, the most frequently observed signs and reported symptoms were diplopia / double vision (43 patients, 84.3%), ocular motility restriction (33 patients, 64.7%), palpebral ecchymosis (16 patients, 31.4%), subconjunctival hemorrhage (8 patients, 15.7%), infraorbital nerve paresthesia (7 patients, 13.7%), and enophthalmos (6 patients, 11.8%) (Table 2).

**Table 2:** Observed clinical signs and reported symptoms at diagnosis of pure orbital fractures in the study population

Clinical signs and symptoms	No. of patients	% of 51
Diplopia (double vision)	43	84.3%
Ocular motility restriction	33	64.7%
Palpebral ecchymosis	16	31.4%
Subconjunctival Hemorrhage	8	15.7%
Infraorbital nerve paresthesia	7	13.7%
Enophthalmos	6	11.8%
Eyelid laceration	2	3.9%
Decreased visual acuity	1	2.0%
Hemorrhagic chemosis	1	2.0%
Subcutaneous emphysema	1	2.0%
Vitreous hemorrhage	1	2.0%
Others	4	-

The timing of intervention from trauma to surgery is reported in Table 3, that highlights that more than two thirds (68.6%) of cases were surgically treated within the first two days from injury.

**Table 3:** Surgical treatment of pure orbital fractures in the study population

	Number of patients	% of 51 patients
**Days from trauma to surgery**		
1st day after trauma	25	49.0%
2nd day	10	19.6%
3rd day	2	3.9%
4th day	-	-
5th day	1	2.0%
6th day	2	3.9%
7th day	2	3.9%
8th day	2	3.9%
9th day	2	3.9%
10th day	-	-
11th day	-	-
12th day	2	3.9%
Other / not specified	3	-
**Indication for surgery**		
Soft tissue entrapment	37	72.5%
Muscle entrapment	35	68.6%
Malglobus (≥ 2 mm of enophtalmos or hypophtalmos)	9	17.6%
**Type of surgical access/approach**		
Lower transconjunctival incision without lateral canthotomy and cantholysis	30	58.8%
Subciliar incision	11	21.6%
Subtarsal lower eyelid (mid lower eyelid) incision	7	13.7%
Lower transconjunctival incision with lateral canthotomy and cantholysis	1	2.0%
Contextual-wound approach	1	2.0%
Other approaches	1	2.0%
**Type of orbital floor reconstruction**		
Shapeable titanium mesh	20	39.2%
Resorbable implant	15	29.4%
No material	6	11.8%
Customized milled titanium mesh	3	5.9%
Prebent titanium mesh	3	5.9%
Porous polyethylene (PPE) sheet (Medpor/ Omnipore)	1	2.0%
Polypropylene (PP) mesh	1	2.0%
Other materials	2	3.9%

The most frequently reported indication for surgery was soft tissue (other than muscle) entrapment (72.5% of cases), together with muscle entrapment in 68.6% of cases (Table 3). Some patients had more than ore indications for surgery.

Several types of surgical approaches were performed (Table 3), with most patients (30 subjects, 58.8%) undergoing lower transconjunctival incision without lateral canthotomy and cantholysis, followed by 21.6% of patients undergoing subciliar incision, and 13.6% of patients subtarsal lower eyelid incision.

As for the used materials for orbital floor reconstruction, in 39.2% of cases (20 patients) a shapeable titanium mesh was used, followed by resorbable implants (15 patients, 29.4%) (Table 3).

Among resorbable implants, in 12 cases polydioxanone sheets were applied, whereas in the remaining 3 patients poly-L/DL-lactide implants were used.

The immediate postoperative presence of diplopia was diagnosed in 25 patients (49.0%) (Table 4). Immediate further postoperative complications after surgery (that had not been diagnosed before surgery) were observed in 7 subjects (13.7%), including infraorbital nerve paresthesia (4 subjects).

**Table 4:** Treatment outcomes of 51 minor patients with pure orbital fractures

**Length of hospital stay (days)**		
mean	2.63 (SD 2.2)	
median	2	
range	1–12	
	**n total**	**% of 51**
**Immediate post-operative presence of diplopia**		
Yes	25	49.0%
**Immediate postoperative complications after surgery**		
Yes	7	13.7%
**Specific immediate postoperative complications after surgery**		
Infraorbital nerve paresthesia	4	7.8%
Decreased visual acuity	-	-
Subconjuctival hemorrhage	-	-
Mild exophthalmos	-	-
Ocular motility restriction	-	-
Other complications	3	-
**Persistent postoperative disturbances / complications 6 months after surgery**		
Yes	6	11.8%
**Specific persistent postoperative disturbances / complications 6 months after surgery**		
Diplopia	3	5.9%
Infraorbital nerve paresthesia	2	3.9%
Enophthalmos	2	3.9%

In 6 patients (11.8%) one or more persistent postoperative disturbances were found six months postoperatively, including diplopia in 3 subjects (5.9%), infraorbital nerve paresthesia in 2 patients (3.9%), and enophthalmos in 2 patients (3.9%) (Table 4).

The 3 patients that still presented diplopia at 6 months follow-up after surgery had the following characteristics: were all boys; were 11, 14 and 16 years old respectively; they all presented isolated orbital floor fracture; they all presented diplopia at diagnosis preoperatively; they all had been surgically treated within the day of trauma; they all underwent surgery because of muscle entrapment; one patient underwent a surgical approach by lower transconjunctival incision without lateral canthotomy and cantholysis was performed, whereas the other two patients underwent subciliar incision; the reconstruction of the orbital floor was performed by different materials in each case (porous polyethylene sheet in a case, resorbable polydioxanone sheet in a case, resorbable poly-L/DL-lactide implant in a case).

Statistically, due to the low numerosity of the study population, no statistically significant association could be found between the presence of diplopia at diagnosis as well as the presence of further signs or symptoms at diagnosis, the timing of intervention after trauma, the type of surgical approach, or the type of orbital reconstruction and the presence of persistent postoperative disturbances were found at 6 months follow up after surgery (*p* > 0.05).

## Discussion

An appropriate diagnosis and accurate management of isolated pediatric orbital walls fractures are fundamental, although it may reveal to be extremely challenging.

The low numerosity of this specific type of fracture make it difficult, if not impossible, to draw conclusions and to elaborate evidence-based guidelines as for its management [1–10].

To this aim, this multicenter study population may contribute to the current knowledge.

As for epidemiology, the present study population of 51 juvenile patients confirmed the predominance of male gender among young patients with pediatric isolated orbital walls fractures. Sports accidents are the most important etiological factor.

The majority (84.3%) of patients presented with an isolated orbital floor fracture, whereas solely 9.8% presented with a combined orbital floor and orbital medial wall fracture, and 5.9% with an orbital medial wall fracture.

Restricted extraocular motility and diplopia are common clinical findings in orbital entrapment fractures. In this multicenter study, diplopia was present in 84.3% and motility restriction in 64.7% of cases, underscoring their role as the most frequent and clinically important symptoms in isolated orbital floor fractures in minors. These findings should be actively sought in pediatric patients with ocular trauma, as well as in those presenting with features of the oculocardiac reflex or unexplained nausea.

Anyway, although clinical examinations are fundamental, it may become challenging in pediatric patients with limited compliance or those with other injuries to achieve sufficient information about visual eye symptoms. In cases of impaired clinical assessment, computed tomography (CT) scans may be extremely useful, although the incarceration of orbital soft tissues in orbital fractures can be missed by radiological assessment [1–5, 8–10].

The long-term morbidities associated with soft tissue incarceration or scarring represent a significant challenge for surgeons and for patients, and their management may require the use of prism glasses and, in more severe cases, strabismus surgery. To prevent long-term functional impairments, prompt diagnosis, timely surgical intervention combined with atraumatic handling of soft tissues is required.

In the present study, a complete resolution of diplopia and while ocular motility restriction was obtained in more than 94% of pediatric patients following the surgical therapy of isolated orbital walls fractures despite a wide timing of intervention from trauma to surgery. More than two thirds (68.6%) of cases were surgically treated within the first two days from trauma.

As aforementioned, the only 3 patients that still presented diplopia at 6 months follow-up after surgery had several different characteristics. They all presented diplopia at diagnosis preoperatively and they all had been surgically treated within the day of injury. These findings suggest that surgical timing was individualized; given the variability in symptoms and soft tissue entrapment, expert judgment yields better outcomes than general timing recommendations.

As for surgical approaches, most patients (58.8%) underwent lower transconjunctival incision without lateral canthotomy and cantholysis, followed by 21.6% undergoing subciliar incision. Instead, as for orbital floor reconstruction, in 39.2% of cases a shapeable titanium mesh was used, followed by resorbable implants (29.4%). The subsequent removal of titanium mesh implants was decided on an individualized case-by-case indication. In fact, the age of the patient, the dimension of the missing floor, and the orbital/midfacial stage of growth were all taken in consideration.

The present multicenter study seems to be in accordance with the results of previous research studies [1–8] assessing orbital walls fractures management in pediatric patients revealing that isolated orbital fractures must be interpreted with caution, above all because of the low numerosity of the involved young study populations together with the multiple different features that any surgeon has to assess and consider in order to plan and perform the best individualized treatment option.

The results of this multicenter study do not, however, exclude the importance of early intervention within 24–48 h after injury, but rather emphasize careful assessment and a reasoned treatment decision that should take into consideration each patient’s individual needs.

Limitations of the present study include its retrospective design, the possibility of treatment-selection bias, and the absent possibility of retrieving the exact fracture and soft tissue involvement extent. Nevertheless, the multicenter study population may have reduced the selection bias typical of single center studies regarding orbital walls fractures.

## Conclusion

The rarity of pure orbital wall fractures in pediatric patients makes it difficult to establish uniform evidence-based guidelines for their management. These injuries must also be recognized promptly, as surgical intervention may be required at a very early stage. Facial surgeons should be aware of the diagnostic and therapeutic challenges they present, with clinical assessment guiding appropriate treatment decisions taking into consideration an individualized choice of the surgical approach and materials. Nevertheless, the retrospective design of the present study together with the possibility of treatment-selection bias do not allow us to draw definitive conclusions.

## Data Availability

No datasets were generated or analysed during the current study.
